# Iodide of Lime a Substitute for Iodide of Potassium

**Published:** 1863-08

**Authors:** James R. Nichols


### IODIDE OF LIME A SUBSTITUE FOR IODIDE OF POTASSIUM.

BY JAMES R. NICHOLS, M. D.

The "Iodide of Lime," first introduced in 1855, by Dr. Puddock, a distinguished London physician, has been rapidly gaining favor among English practitioners, as a remedy of great value. It is used in those cases where iodide of potassium is indicated, with more marked effects than usually attend the use of that salt. The success attending its use has led us to prepare the article with much care. The lime and iodine are held together by a very feeble affinity, and the salt will not admit of exposure without evolving free iodine. The *solution* is a colorless and almost tasteless liquid, and remains permanent although long kept and exposed to the air.

Each drachm of the salt contains eight and a half grains of iodine; and each fluid ounce of the solution contains half a grain of iodine. The iodine *in the solution* exists in the form of iodide of calcium and iodate of lime, thus:—$6CaO \bowtie 61 = 5CaI \bowtie CaO, IO_5$. Acids decompose the solution, and free the iodine; hence the utility of this form for the administration of iodine, probably in the state of an oxide. The iodide of lime is superior to iodide of potassium in several particulars, as—

1—The smallness of the dose, and the minute state of its atomic division. 2—In not passing off so quickly through the kidneys. 3—In its ready combination with the blood and tissues, manifested by its alterative effects. 4—In being nearly tasteless, and therefore readily taken by children. 5—In being much less expensive. 6—In not producing either gastro-enteritio or vesical irritation.

Iodide of potassium is an expensive remedy; and on that account many physicians refrain from using it. The iodide of lime being found to be more efficacious and much cheaper, will doubtless be substituted for it to a great extent. It has been used in England with much success in throat diseases, in morbid conditions of the general system, in scrofulous affecti ›ns, in intractable cases of neuralgia, in diseases caused by metalic poisons, etc. At the Bloomsbury Dispensary, England, it has been extensively prescribed for three years with much success.

The dose of the salt is very small; about one-fourth of a grain given in solution two or three times a day. Of the solution, two or four fluid drachms may be given as often. The small quantity required for a dose renders this preparation *cheaper* than any of the medicinal iodides. The iodide of potassium, at one-half the cost per ounce, given in six-grain doses, is more than *ten times as expensive* as iodide of lime.

Physicians may find it convenient to prepare the contents of an ounce bottle at once. This may be done by simply pouring on to the salt in a tin or porcelain-lined vessel one gallon of boiling water. Some carbonate of lime falls to the bottom, and the solution soon becomes clear and ready for use. It can be kept in a cool place for several months.

The SOLUTION should always be used in preference to the salt.

For the numerous class of *eruptive affections,* chronic or acute, in children or adults, nothing can exceed the beneficial effects of iodide of lime. It may be combined with sarsaparilla, or any other of the vegetable alterative or tonic agents.

